# Supplementary material for: Favorable Effect of Pemafibrate on Insulin Resistance and β-Cell Function in Subjects with Type 2 Diabetes and Hypertriglyceridemia: A Subanalysis of the PARM-T2D Study
Source: Pharmaceutics. 2023 Jun 27;15(7):1838. doi: 10.3390/pharmaceutics15071838 (PMC10386707; doi:10.3390/pharmaceutics15071838)
Supplement: Supplementary file 1 [file pharmaceutics-15-01838-s001.zip › Supplementary Figure.pdf]

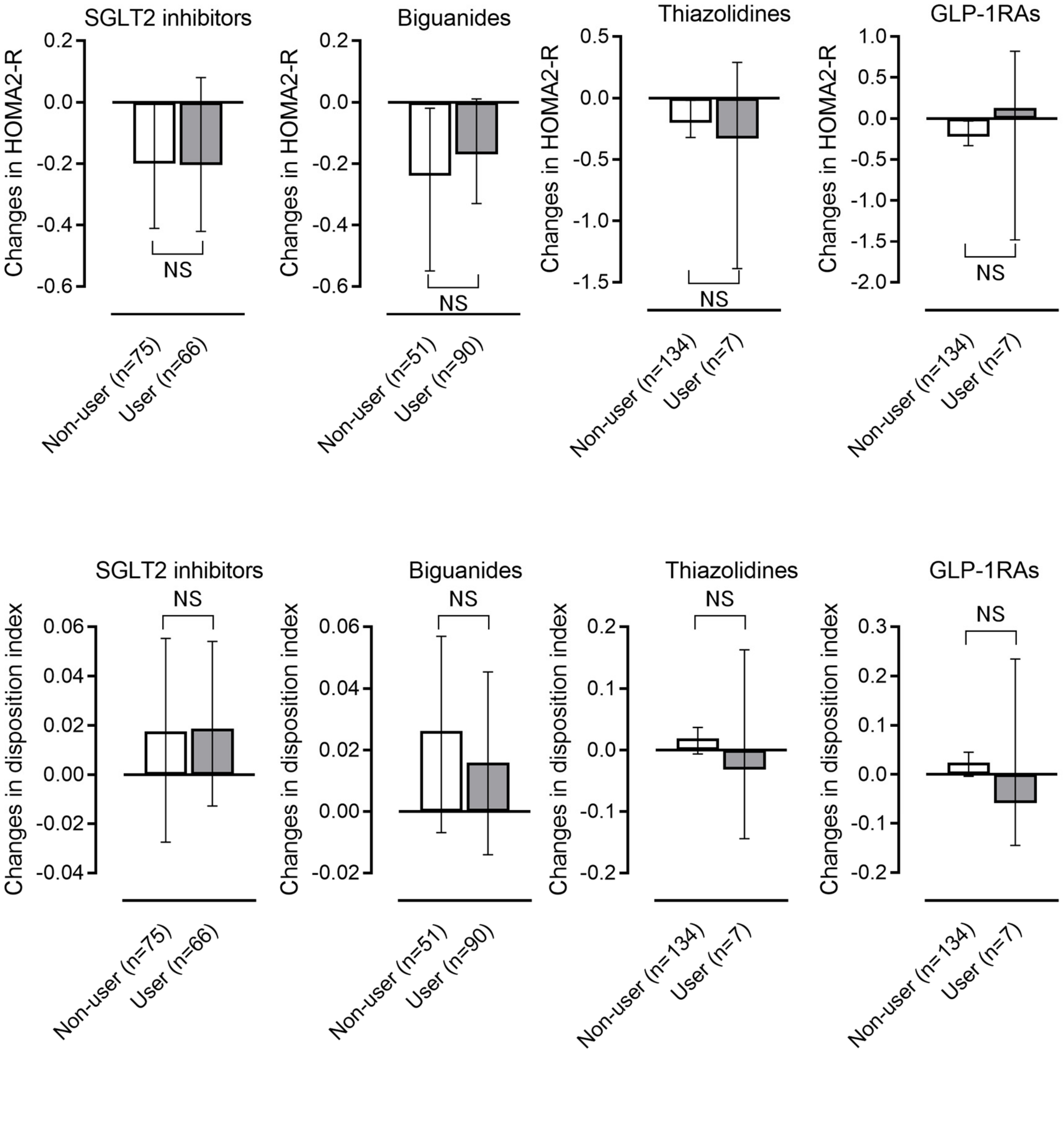

Supplementary Figure S1. Changes in HOMA2-R and disposition by concomitant medication for diabetes in the pemafibrate group from baseline. Data are shown as the median change (95% confidence interval). GLP-1RAs, glucagon-like peptide-1 receptor agonists; SGLT2, sodium glucose cotransporter 2; NS, not significant.
