# Supplementary material for: Favorable Effect of Pemafibrate on Insulin Resistance and β-Cell Function in Subjects with Type 2 Diabetes and Hypertriglyceridemia: A Subanalysis of the PARM-T2D Study
Source: Pharmaceutics. 2023 Jun 27;15(7):1838. doi: 10.3390/pharmaceutics15071838 (PMC10386707; doi:10.3390/pharmaceutics15071838)
Supplement: Supplementary file 1 [file pharmaceutics-15-01838-s001.zip › Supplementary Tables.pdf]

**Table S1.** Differences in baseline characteristics between the improved group and the deteriorated group based on changes in HOMA2-R in the pemafibrate group during the 52-week study period.

| **Variable** | **Improved group (n=83)** | **Deteriorated group (n=58)** | ***P*-value** |
|---|---|---|---|
| Age (years) | 60.1 ± 12.7 | 60.1 ± 11.7 | 0.979 |
| Female sex | 27 (32.5) | 18 (31.0) | 1.000 |
| BMI (kg/m$^2$) | 27.4 ± 4.2 | 27.2 ± 3.8 | 0.718 |
| FPG (mg/dL) | 140.1 ± 32.1 | 128.3 ± 26.3 | 0.023 |
| HbA1c (%) | 7.02 ± 0.84 | 6.79 ± 0.68 | 0.085 |
| Triglyceride (mg/dL) | 180 (137, 249) | 163 (130, 218) | 0.226 |
| HDL-C (ng/mL) | 52.6 ± 12.4 | 52.1 ± 11.2 | 0.825 |
| AST (IU/L) | 32.7 ± 17.5 | 29.8 ± 12.8 | 0.281 |
| ALT (IU/L) | 31 (21, 48) | 29 (19, 43) | 0.404 |
| γ-GTP (IU/L) | 45 (31, 84) | 34 (22, 56) | 0.007 |
| eGFR (mL/min/1.73m$^2$) | 71.1 ± 20.1 | 67.4 ± 15.4 | 0.941 |
| HOMA2-R | 2.30 (1.74, 3.14) | 1.75 (1.32, 2.37) | <0.001 |
| HOMA2-β | 74.4 (61.1, 90.6) | 76.5 (53.1, 97.7) | 0.695 |
| Fibrates | 28 (33.7) | 24 (41.4) | 0.379 |
| Statins | 60 (72.3) | 39 (67.2) | 0.576 |
| α-glucosidase inhibitors | 0 (0) | 2 (3.4) | 0.168 |
| Biguanides | 50 (60.2) | 40 (69.0) | 0.373 |
| DPP-4 inhibitors | 35 (42.2) | 31 (53.4) | 0.234 |
| GLP-1 receptor agonists | 3 (3.6) | 4 (6.9) | 0.446 |
| SGLT2 inhibitors | 37 (44.6) | 29 (50.0) | 0.608 |
| Thiazolidines | 4 (4.8) | 3 (5.2) | 1.000 |

Data are shown as mean ± SD, median (25% percentile, 75% percentile), or number (%). *P*-values for pemafibrate group versus control group were obtained using Student's *t*-test, the Mann–Whitney test, or Fisher's exact test. BMI, body mass index; FPG, fasting plasma glucose; HbA1c, glycated hemoglobin; HDL-C, high-density lipoprotein cholesterol; AST, aspartate aminotransferase; ALT, alanine aminotransferase; ALP, alkaline phosphatase; γ-GTP, γ-glutamyl transpeptidase; eGFR, estimated glomerular filtration rate; HOMA, Homeostatic model assessment; DPP-4, dipeptidyl peptidase-4; GLP-1, glucagon-like peptide-1; SGLT2, sodium glucose cotransporter 2.

**Table S2.** Differences in baseline characteristics between the improved group and the deteriorated group based on changes in disposition index in the pemafibrate group during the 52-week study period.

| **Variable** | **Improved group (n=79)** | **Deteriorated group (n=62)** | ***P*-value** |
|---|---|---|---|
| Age (years) | 60.0 ± 12.7 | 60.2 ± 11.8 | 0.930 |
| Female sex | 30 (38.0) | 15 (24.2) | 0.102 |
| BMI (kg/m$^2$) | 27.4 ± 4.2 | 27.3 ± 3.8 | 0.920 |
| FPG (mg/dL) | 142.6 ± 34.8 | 126.8 ± 19.8 | <0.001 |
| HbA1c (%) | 7.08 ± 0.88 | 6.72 ± 0.59 | 0.007 |
| Triglyceride (mg/dL) | 171 (132, 237) | 170 (138, 232) | 0.935 |
| HDL-C (ng/mL) | 52.5 ± 11.6 | 52.2 ± 12.3 | 0.888 |
| AST (IU/L) | 32.1 ± 16.9 | 30.8 ± 14.2 | 0.640 |
| ALT (IU/L) | 30 (21, 54) | 30 (20, 41) | 0.443 |
| γ-GTP (IU/L) | 44 (24, 84) | 35 (25, 64) | 0.377 |
| eGFR (mL/min/1.73m$^2$) | 71.7 ± 20.8 | 66.8 ± 14.2 | 0.112 |
| HOMA2-R | 2.30 (1.66, 3.09) | 1.97 (1.39, 2.59) | 0.033 |
| Disposition index | 0.33 (0.25, 0.44) | 0.38 (0.30, 0.49) | 0.004 |
| Fibrates | 23 (29.1) | 29 (46.8) | 0.036 |
| Statins | 58 (73.4) | 41 (66.1) | 0.360 |
| α-glucosidase inhibitors | 0 (0) | 0 (0) | 1.000 |
| Biguanides | 50 (63.3) | 40 (64.5) | 1.000 |
| DPP-4 inhibitors | 35 (44.3) | 31 (50.0) | 0.610 |
| GLP-1 receptor agonists | 1 (1.3) | 6 (9.7) | 0.446 |
| SGLT2 inhibitors | 37 (46.8) | 29 (46.8) | 1.000 |
| Thiazolidines | 3 (3.8) | 4 (6.5) | 0.699 |

Data are shown as mean ± SD, median (25% percentile, 75% percentile), or number (%). *P*-values for pemafibrate group versus control group were obtained using Student's *t*-test, the Mann–Whitney test, or Fisher's exact test. BMI, body mass index; FPG, fasting plasma glucose; HbA1c, glycated hemoglobin; HDL-C, high-density lipoprotein cholesterol; AST, aspartate aminotransferase; ALT, alanine aminotransferase; ALP, alkaline phosphatase; γ-GTP, γ-glutamyl transpeptidase; eGFR, estimated glomerular filtration rate; HOMA, Homeostatic model assessment; DPP-4, dipeptidyl peptidase-4; GLP-1, glucagon-like peptide-1; SGLT2, sodium glucose cotransporter 2.

**Table S3.** Changes from baseline in key endpoints for the participants.

| | Pemafibrate (n=141) | Control (n=138) | |
|---|---|---|---|
| **Variables** | **Mean change at 52 wks** | **Mean change at 52 wks** | ***P*-value** |
| Body mass index (kg/m$^2$) | 0.0 (−0.1 to 0.2) | −0.1 (−0.2 to 0.1) | 0.489 |
| Total cholesterol (mg/dL) | −6.4 (−10.4 to −2.4) ** | −1.1 (−5.4 to 3.1) | 0.076 |
| Triglycerides (mg/dL) | −46 (−60 to −30) *** | −14 (−20 to 0) * | < 0.001 |
| HDL-cholesterol (mg/dL) | 2.0 (0.3 to 3.8) * | −0.2 (−1.2 to 0.9) | 0.035 |
| AST (IU/L) | −3.5 (−5.4 to −1.6) *** | 0.7 (−1.4 to 2.7) | 0.003 |
| ALT (IU/L) | −6 (−9 to −4) *** | −1 (−2 to 0) | < 0.001 |
| γ-GTP (IU/L) | −10 (−14 to −5) *** | −1 (−2 to 1) | < 0.001 |
| eGFR (mL/min/1.73m$^2$) | 2.5 (0.6 to 4.4) | −0.7 (−2.2 to 0.8) | 0.009 |

Data are shown as mean or median change (95% confidence interval). *P*-values for pemafibrate group versus control group were obtained using Student's *t*-test, the Mann–Whitney test. * $P < 0.05$, ** $P < 0.01$, *** $P < 0.001$ vs baseline. AST, aspartate aminotransferase; ALT, alanine aminotransferase; γ-GTP, γ-glutamyl transpeptidase; eGFR, estimated glomerular filtration rate.

**Table S4.** Relationships between changes in indices for glucose metabolism and changes in metabolic pa-rameters in the pemafibrate group evaluated by multiple linear regression analysis.

| ΔHOMA-2R | Regression coefficients | 95% confidence interval | *P* value |
|---|---|---|---|
| ΔHDL-C | −0.058 | −0.101, −0.022 | 0.003 |
| Δγ-GTP | 0.004 | −0.001, 0.009 | 0.113 |
| ΔALT | 0.002 | −0.011, 0.015 | 0.772 |
| **ΔDI** | **Regression coefficients** | **95% confidence interval** | ***P* value** |
| ΔTG | −0.00022 | −0.00038, −0.00005 | 0.011 |
| ΔBMI | −0.03373 | −0.06252, −0.00494 | 0.022 |
| Δγ-GTP | −0.00047 | −0.00112, 0.00019 | 0.164 |
| ΔeGFR | −0.00138 | −0.00363, 0.00086 | 0.226 |

Multiple linear regression was adjusted for age, sex, high-density lipoprotein cholesterol (HDL-C), γ-glutamyl transpeptidase (γ-GTP) and alanine aminotransferase (ALT) for homeostatic model assessment (HOMA)-2R, and age, sex, triglyceride (TG), body mass index (BMI), γ-GTP, and estimated glomerular filtration rate (eGFR) for disposition index (DI).
